# Over-Expression of hNGF in Adult Human Olfactory Bulb Neural Stem Cells Promotes Cell Growth and Oligodendrocytic Differentiation

**DOI:** 10.1371/journal.pone.0082206

**Published:** 2013-12-19

**Authors:** Hany E. S. Marei, Asmaa Althani, Nahla Afifi, Ahmed Abd-Elmaksoud, Camilla Bernardini, Fabrizio Michetti, Marta Barba, Mario Pescatori, Giulio Maira, Emanuela Paldino, Luigi Manni, Patrizia Casalbore, Carlo Cenciarelli

**Affiliations:** 1 Department of Cytology and Histology, Faculty of Veterinary Medicine, Mansoura University, Mansoura, Egypt; 2 College of Arts & Sciences, Health Sciences Department, Qatar University, Doha, Qatar; 3 Department of Anatomy, Faculty of Medicine, Ain Shams University, Cairo, Egypt; 4 Institute of Anatomy and Cell Biology, Università Cattolica del S. Cuore, Roma, Italy; 5 Department of Surgery, Erasmus MC, Rotterdam, The Netherlands; 6 Institute of Neurosurgery, Università Cattolica del Sacro Cuore, Roma, Italy; 7 Institute of Cell Biology and Neurobiology, National Research Council of Italy, Roma, Italy; 8 Institute of Translational Pharmacology, National Research Council of Italy, Roma, Italy; Kaohsiung Chang Gung Memorial Hospital, Taiwan

## Abstract

The adult human olfactory bulb neural stem/progenitor cells (OBNC/PC) are promising candidate for cell-based therapy for traumatic and neurodegenerative insults. Exogenous application of NGF was suggested as a promising therapeutic strategy for traumatic and neurodegenerative diseases, however effective delivery of NGF into the CNS parenchyma is still challenging due mainly to its limited ability to cross the blood–brain barrier, and intolerable side effects if administered into the brain ventricular system. An effective method to ensure delivery of NGF into the parenchyma of CNS is the genetic modification of NSC to overexpress NGF gene. Overexpression of NGF in adult human OBNSC is expected to alter their proliferation and differentiation nature, and thus might enhance their therapeutic potential. In this study, we genetically modified adult human OBNS/PC to overexpress human NGF (hNGF) and green fluorescent protein (GFP) genes to provide insight about the effects of hNGF and GFP genes overexpression in adult human OBNS/PC on their in vitro multipotentiality using DNA microarray, immunophenotyping, and Western blot (WB) protocols. Our analysis revealed that OBNS/PC-GFP and OBNS/PC-GFP-hNGF differentiation is a multifaceted process involving changes in major biological processes as reflected in alteration of the gene expression levels of crucial markers such as cell cycle and survival markers, stemness markers, and differentiation markers. The differentiation of both cell classes was also associated with modulations of key signaling pathways such MAPK signaling pathway, ErbB signaling pathway, and neuroactive ligand-receptor interaction pathway for OBNS/PC-GFP, and axon guidance, calcium channel, voltage-dependent, gamma subunit 7 for OBNS/PC-GFP-hNGF as revealed by GO and KEGG. Differentiated OBNS/PC-GFP-hNGF displayed extensively branched cytoplasmic processes, a significant faster growth rate and up modulated the expression of oligodendroglia precursor cells markers (PDGFRα, NG2 and CNPase) respect to OBNS/PC-GFP counterparts. These findings suggest an enhanced proliferation and oligodendrocytic differentiation potential for OBNS/PC-GFP-hNGF as compared to OBNS/PC-GFP.

## Introduction

Exogenous application of nerve growth factor (NGF) for the treatment of traumatic and neurodegenerative insults is a promising therapeutic strategy. NGF enhances the survival of cholinergic neurons in basal forebrain in rats [1–3] and primates [4–7], and phase-I clinical trial of NGF gene therapy for Alzheimer’s disease (AD) provided promising data [8,9].

Effective delivery of NGF into the CNS parenchyma is still challenging due mainly to its limited ability to cross the blood–brain barrier, and intolerable side effects (pain, aberrant sympathetic, sensory neurite sprouting, and weight loss) if administered into the brain ventricular system Intranasal administration of NGF rescued recognition memory deficits in an anti-NGF transgenic mouse model which shows typical features of AD [10–12]. Previous studies using adenoviral neurotrophic gene transfer indicate that it provided an effective tool for the delivery of potentially therapeutic proteins to the injured or diseased spinal cord [13,14].

An effective method to ensure delivery of NGF into the parenchyma of CNS is the genetic modification of cells to overexpress NGF gene(s). In this regard, engraftments of cells that secrete NGF promote the growth of host spinal axons after injury [15] and protect cholinergic neurons from degeneration in chemical lesions [16,17] or aged brain [18–20]. Transplantation of fibroblasts encoding NGF gene in the primate brain rescued degenerating cholinergic neurons, and reduce degree of cognitive decline [20].

Identification of suitable cellular carriers for therapeutic transgenes is a crucial prerequisite for successful application of in vivo gene transfer to the CNS. In adult humans, neural stem/progenitor cells (NS/PC) have successfully been isolated from the olfactory bulb (OB), which therefore represents an accessible source of neural precursors for transplantation-based therapy that avoids the ethical issues raised by the use of human embryos, and provide an innovative autotransplantation strategy for neurodegenerative diseases [21–24] The discovery of a large number of immunoreactive tyrosine hydroxylase structures in the olfactory bulbs of elderly humans [22] suggests that the olfactory bulb is a source for the autotransplantation therapy in Parkinson’s disease.

It has been suggested that the NSCs engrafted at sites of nerve injury promote functional recovery by producing trophic factors such as nerve growth factor (NGF) which induces the survival and regeneration of different neuronal subtypes [25–32]. Transplantation of human NSCs expressing diverse functional genes, especially encoding growth factors, preserves host cells and restored function in animal models of AD, Parkinson’s disease (PD), Huntington’s disease (HD), Amyotrophic Lateral Sclerosis (ALS), stroke and spinal cord injury (SCI) [33–40]. In our previous work, we have studied the gene expression profile of wild type adult human OBNS/PC in comparison to embryonic ones and demonstrated the existence of distinct signaling pathways and epigenetic control between them [41,42]. In this study, we genetically modified adult human OBNS/PC to overexpress human NGF (hNGF) and green fluorescent protein (GFP) genes, which are common genes used to trace engrafted NSCs and to enhance their therapeutic potential against traumatic and neurodegenerative diseases [44,45]. Wether or not such genetic alterations will have an effect on their *in vitro* proliferation and differentiation potential is still not clear. Therefore, the primary objective of this study was to provide insight about the effects of hNGF and GFP genes over expression in adult human OBNS/PC on their *in vitro* proliferation and differentiation potential as revealed from modulations in their target genes and corresponding pathways during their proliferation and differentiation using DNA microarray, immunophenotyping and Western blot protocols. The present study reports the up-regulation of immature oligodendrocyte markers such as PDGFRα, NG2 and CNPase proteins in differentiated OBNS/PC-GFP-hNGF, while reveals a down modulation of the same markers in differentiated OBNS/PC-GFP. These findings suggest an enhanced proliferation and oligodendrocytic differentiation potential for OBNS/PC-GFP-hNGF as compared to OBNS/PC-GFP.

## Materials and Methods

### Isolation and Culturing of Human Olfactory Bulb NS/PC Ethical statement

Written informed consent was obtained and all patients were fully aware of the scope and aims of work. This procedure was obtained in the past years between 2006 and 2008. The informed consent was requested by the PLoS one journal to publish our article: Tumorigenic Potential of Olfactory Bulb-Derived Human Adult Neural Stem Cells Associates with Activation of TERT and NOTCH1. Patrizia Casalbore et al. February 2009 | Volume 4 | Issue 2 | e4434, and our second article: Marei HE, Ahmed AE, Michetti F, Pescatori M, Pallini R, Casalbore P, Cenciarelli C, Elhadidy M. Gene expression profile of adult human olfactory bulb and embryonic neural stem cell suggests distinct signaling pathways and epigenetic control. PLoS One. 2012; 7(4):e33542. doi: 10.1371/journal. pone.0033542. Epub 2012 Apr 2. Procedures for collection and isolation of adult human OBNS/PC were approved by the Ethical Committee of the Catholic University, Italy.

We have used the same clinical materials, and protocol used in our previous papers to isolate, and culture the human OBNS/PC [41,42]. In brief, the OB cells were harvested from adult patients undergoing craniotomy at the Institute of Neurosurgery, Catholic University, Rome [22]. Immediately after removal, the OBs were dissociated in Papain 0.1% (Sigma-Aldrich, St. Louis, MO) for 30 minutes at 37^º^C. Dissociated cells were cultured in the presence of human recombinant EGF (20 ng/ml; PeproTech, Rocky Hill, NJ), human recombinant bFGF (10 ng/ml; PeproTech), and LIF (20 ng/ml; Immunological Sciences, Rome, Italy) in DMEM/F12 (1:1) serum-free medium (Invitrogen, Carlsband, CA) containing L glutamine 2 mM, glucose 0.6%, putrescine 9.6 ug/ml, progesterone 0.025 mg/ml, sodium selenite 5.2 ng/ml, insulin 0.025 mg/ml, apo-transferrin sodium salt 0.1 mg/ml, sodium bicarbonate 3 mM, Hepes 5 mM, BSA 4 mg/ml, heparin 4 ug/ml. Primary neurospheres were dissociated with Accutase (Invitrogen), serially diluted and plated one cell per mini-well onto 96-well plates. Mini-wells containing one single cell were marked after microscopic confirmation and assessed for secondary neurosphere generation after two weeks. Secondary neurospheres were subsequently dissociated, plated at the density of 10^3^ cells/cm^2^ in serum-free medium containing EGF and bFGF, and passaged up to P30. Between P7 and P10 OBNS/PC were infected with lentivirus transducing hNGF. Differentiation assays were performed within 5 days after plating on Matrigel coated glass coverslips in the absence of EGF and bFGF and in the presence of 1% fetal calf serum (Hyclone) supplemented with 100µM 3'–5'-cyclic adenosine monophosphate (cAMP), 1µM all-trans retinoic acid (Sigma Aldrich) and 30nM triiodothyronine (T3) (Sigma Aldrich) [21].

### Construction of lentiviral expression vectors

pLentiTrident1, the lentiviral vector backbone, was purchased by Cistronics Cell Technology GmbH (Zurich, Switzerland). The construction of pLentiTrident(CMV)::EGFP has been described [43], hereby it is briefly summarized: the PCR fragment coding EGFP was derived from pEGFP-C1 (Clontech). The pLentiTrident1-CMV::EGFP::Ires::Neo/Kana (GFP-vector) was realized by cloning Neomycin/Kanamycin open reading frame digested with Not1-Cla1 enzymes and ligated downstream to the first IRES (internal ribosomal entry site) of pLentiTrident1-CMV::EGFP. The pLentiTrident1-CMV::EGFP::Ires::Neo/Kana::Ires::hNGF (or hNGF-vector) was obtained by PCR of the pro-peptide hNGF cDNA derived from pCMVSPORT 6-hNGF. Subsequently, the PCR product digested with Pac1 and Swa1 enzymes was ligated downstream to the second IRES of pLentiTrident1-CMV::EGFP::Ires::Neo/Kana. The hNGF and EGFP PCR products were sequenced to verify accuracy (MWG Biotech).

### Transfection and infection

Human embryonic kidney (HEK)-293T cells in log-phase growth were transiently transfected, using standard LipofectAmine reagent (Invitrogen), with either GFP-Vector or hNGF-vector plus helper plasmids to produce virions [43]. Media containing virions were collected two days after cell transfection and transferred directly onto OBNS/PC. Lentiviral infection was performed in the presence of polybrene solution at 8 mg/ml (Sigma-Aldrich). Antibiotic G418 (Euroclone) was added to the cells at 400mg/ml over time for OBNS/PC selection and maintenance.

### Enzyme linked immunosorbent assay (ELISA)

hNGF released by the group of control OBNS/PC-GFP and OBNS/PC-GFP-hNGF was measured by two-site ELISA kit (R&D System Kit) as described previously [43]. For hNGF detection conditioned media were collected from the cell cultures seeded in triplicates in either proliferation or differentiation conditions. Cellular pellets were also collected from each group respectively for protein determination. Optical absorbance was read at 450 nm by a microplate reader. The readouts expressed in pg/ml were adjusted by the total amount of cellular protein contents. P values <0.05 (*) and <0.001 (***) were considered statistically significant

### Affymetrix Genechip hybridizations and analysis of expression data

Total RNA was extracted using the TriZol reagent (TriZol, Invitrogen, Carlsbad, CA, USA) and further purified using the RNAeasy mini kit following the RNA cleanup protocol as indicated by the manufacturer (Qiagen, Valencia, CA, USA). RNA purity was assessed by spectrophotometric analysis and integrity by microfluidic molecular sizing using the Bioanalyzer 2100 (Agilent). Samples with RIN (RNA Integrity Number) <8 were discarded and not used in the gene expression analysis. One microgram of total RNA was converted in cRNA and labeled as described in the Affimetrix GeneChip® Whole Transcript (WT) Sense Target Labeling Assay Manual. We made use of the Affymetrix technology to analyze the expression of 32,020 RefSeq coding transcript with well-established annotations, using the Human Gene 1.0 ST Array (Affymetrix, Santa Clara, CA, USA) following standard protocols. Hybridised Genechips were processed] and experiments were performed in triplicate.

Gene expression measures were extracted and normalized from CEL files using the RMA algorithm implemented in Affymetrix Expression Console. The same software was used for QC analysis of the genechips. Statistical analysis and visualization of gene expression data was performed using BRB-ArrayTools, developed by R. Simon and the BRB ArrayTools Development Team. To define the gene expression changes induced by NGF over-expression and cell differentiation, we computed the probability of genes being differentially expressed between the classes using the random variance t test as implemented in BRBArrayTools. Genes were considered statistically significant if p<0.001. *Per gene* false discovery rate was computed using the method of Benjamini and Hochberg [46]. Significant genes were clustered and displayed as heatmap using the clustering tool in BRB ArrayTools.

### Data analysis

#### Gene Ontology Analysis

Gene ontology analysis was conducted according to [46–50]. For each GO group we computed the number n of genes represented on the microarray in that group, and the statistical significance pi value for each gene i in the group. We considered a GO category significantly differentially regulated if either significance level was less than 0.01.

The evaluation of which Gene Ontology classes are differentially expressed between pre- and post treatment samples was performed using a functional class scoring analysis as described by Pavlidis et al. [49]. The functional class scoring analysis for Gene Ontology classes was performed using BRB-ArrayTools. MATLAB software (v. 7.3) was used for clustering and correlation. Expander software (v. 5.07) [50] was used for the hierarchical clustering of transcripts overexpressed in each stage separately and cell cycle associated transcripts. The STRING database (version 8.1) [51] was used to construct a regulatory network of differentially expressed transcripts. The visualization of networks was performed using Cytoscape (version 2.6.3) [52]. We used BiNGO (a Cytoscape plugin) [53] to find statistically over- or underrepresented Gene Ontology (GO) categories in the biological data as a tool to enrich the analysis of the transcriptome dataset.

#### Functional Annotation and Molecular Network Analysis

Functional annotation of significant genes identified by microarray analysis was searched by the web-accessible program named Database for Annotation, Visualization and Integrated Discovery (DAVID) version 2009, National Institute of Allergy and Infectious Diseases (NIAID), National Institutes of Health (NIH) (david.abcc.ncifcrf.gov) [41,42]. Gene ontology (GO) and KEGG molecular pathway analysis was performed to identify possible enrichment of genes with specific biological themes using both the data set as a whole and then in the individual K-means clusters. DAVID calculates a modified Fishers Exact p-value to demonstrate GO or molecular pathway enrichment, where p-values less than 0.05 after Benjamini multiple test correction are considered to be strongly enriched in the annotation category [54,55].

### Immunocytochemistry

OBNS/PC-derived cell populations finely dissociated with Pasteur pipettes were plated onto Matrigel GFR-coated glass coverslips (BD-Italia) to assay their multipotentiality within 5 days in vitro (DIV) by withdrawal of EGF and bFGF and adding of 1% fetal calf serum (Hyclone, Logan, USA) supplemented with 1µM ATRA, 100µM 3'–5'-cyclic adenosine monophosphate (cAMP), with or w/o 30nM triiodothyronine (T3) and 40ng/ml PDGFAA (all purchased from Sigma Aldrich) [23]. OBNS/PC were fixed in 4% paraformaldehyde and permeablized with 0.2% Triton-100 (last step was not performed for NG2 and O4 immunostaining) and subsequently processed for immunolabeling. The following antibodies were used: anti-glial fibrillary acidic protein (GFAP) rabbit polyclonal (DAKO), anti-tubulin β-isoform III mouse monoclonal, mouse anti-MAP2 (a/b), rabbit anti-pro-oligodendrocytes chondroitin sulphate proteoglycan (NG2) and IgM anti-O4 (all from Chemicon), rabbit anti-Nestin (Millipore). Tetramethyl rhodamine isothiocyanate (TRITC) affinity purified goat anti-rabbit for dual labeling (Chemicon) and fluorescein isothiocyanate (FITC) affinity purified donkey anti-mouse for dual labeling (Chemicon). Cellular nuclei were stained with Hoechst 33258 diluted in PBS (0,2µg/ml; SIGMA). Immunocytochemistry analysis was performed using a fluorescent microscope (Olympus microscope OLYMPUS Bx5 with Spot CCD Camera) and cell were photographed at 400X and 600X magnification with either two filter or three filter sets for detection of immunolabelled cells Differentiated cells percentage was evaluated by counting 100cells/field of six independent fields positive for each specific antibody respect to total stained nuclei.

### Western Blot

OB cells kept in proliferation and differentiation conditions (almost 2 x10^6^ cells) were harvested after 5 Days in vitro (DIV), lysed and sonicated with two pulses of 5 sec with 50% of amplitude (Sonics and Materials, Newtown, CT) in lysis buffer containing: 1% NP-40, 0.01% SDS, 20mM Tris–HCl pH 7.4, 300mM NaCl, 1mM EGTA, 1mMEDTA, 1mM Na _3_VO_4_ and protease inhibitors cocktail (Sigma Aldrich). Equal amount (40–50µg) of total protein extracts, determined using Bio-Rad protein Assay (Bio-Rad, Munchen, Germany), were loaded on NuPAGE Bis-Tris gels (Invitrogen), then transferred on Hybond-P Extra membrane (Amersham Biosciences, GE Healthcare Life Science-Buckinghamshire, UK) and immunoblotted using the following primary antibodies: rabbit anti-p-ERK1/2 and anti-ERK1/2 (Cell Signaling, MA-USA), rabbit anti-p-CREB and mouse anti-CREB (Cell Signaling), mouse anti-p-AKT1 and rabbit anti-AKT1 (Calbiochem), rabbit anti-NeuroD1 and anti-Nurr1 (Millipore), rabbit anti-TH (Cell Signaling), rabbit anti-CyclinD1, anti-p27, anti-Trks, anti-TrkA and anti-Sox2 (all purchased from Santa Cruz-USA), rabbit anti-Oct4 (Millipore), rabbit anti-NICD1 (Cell Signaling), mouse anti-GFAP (Covance), rabbit anti-NG2 and mouse anti-CNPase (Chemicon), goat anti-PDGFRα and mouse anti-β-actin (from SIGMA). After three washing with TBS-T buffer, immuno-reactive proteins were detected using rabbit-anti-mouse, donkey-anti-rabbit and donkey anti-goat horseradish peroxidase-conjugated secondary antibodies directed to the appropriate primary antibodies (Jackson Immunoresearch Laboratories, West Grove, PA). The proteins were then visualized using the chemiluminescence system (Millipore).

The densitometric analysis of protein bands normalized against to β-actin protein levels were performed from three independent experiments using the ImageJ software (NIH, USA).

### MTS assay

We used the CellTiter 96 Aqueous One Solution Reagent (Promega), a cell proliferation colorimetric assay containing a novel tetrazolium compound [3-(4,5-dimethylthiazol-2-yl)-5-(3-carboxymethoxyphenyl)-2-(4-sulfophenyl)-2H-tetrazolium; MTS] and an electron-coupling reagent (phenazine methosulfate). Neurospheres were dissociated into single cells and 10^4^ cells/well plated in triplicate for each group on six-well plates. The day after cells were collected at 0, 1, 2, 3 and 4 DIV in growth medium cells and incubated with 100µl/ml MTS at 37° C for approximately 1 h. The metabolically active cells reduced MTS into a soluble formazan product, the absorbance of which was measured at 490 nm. The absorbance values of the collected samples were subtracted from the background absorbance of medium-only control and expressed as absorbance values. P values <0.05 (*) and <0.001 (***) were considered statistically significant.

### Statistical analysis

Statistical analysis was performed with Prism5 (GraphPad) and Microsoft Office Excel 2007. MTS and ELISA assays were analyzed by Two-way ANOVA and Bonferroni’s post tests. Data are expressed as mean ± standard error of mean (S.E.M) and P values <0.05 (*) and <0.001 (***) were considered statistically significant.

## Results and Discussion

In our previous study, we have deciphered the basal gene expression profile of human OBNS/PC and demonstrated that there were no significant differences in the gene expression profiles of our examined OBNS/PC populations over prolonged period of culture, and that OBNSCs were genetically stable during the examined time scale [41,42]. Here, we carried out microarray gene expression studies to identify the target genes and corresponding pathways in response to overexpression of NGF genes during their proliferation and differentiation. Understanding mechanisms involved in modulation of proliferation and differentiation potential of adult human OBNSC is crucial for enhancement of their therapeutic potentials against a wide range of traumatic and neurodegenerative diseases such as AD, PD among others.

In the present study, the proliferated and differentiated human olfactory bulb neural stem/progenitor cells (OBNS/PC) were infected with lentivirus transducing GFP and GFP-hNGF gene(s). Following lentivirus-mediated of GFP and GFP-hNGF gene (s) transduction, the OBNS/PC-GFP and OBNS/PC-GFP-hNGF were analysed for NGF mRNA and protein expression.

**Figure 1:**
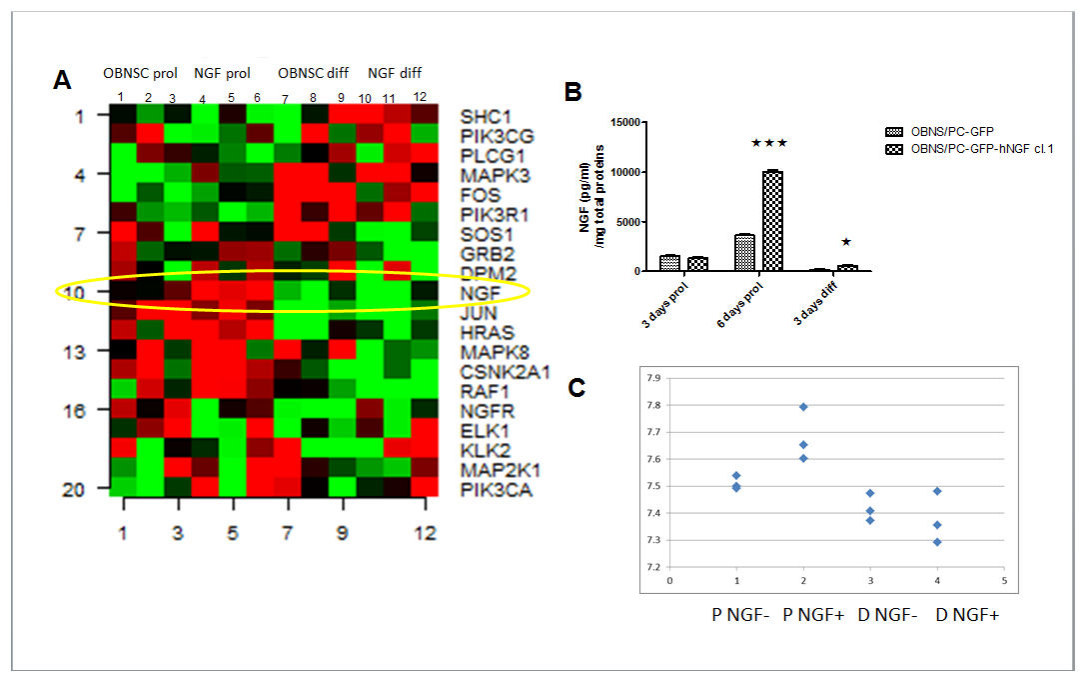
Heatmap of representative cluster transcripts involved in NGF signaling. A. OBNS/PC-GFP proliferation (OBNSC prol.), OBNS/PC-hNGF proliferation (NGF prol.), OBNS/PC-GFP differentiation (OBNSC diff.), and OBNS/PC-GFP-hNGF differentiation (NGF diff). Transcripts that are highly up-regulated are red compared with the down-regulated ones which are green. NGF is up-regulated in OBNS/PC-GFP-hNGF respect to OBNS/PC-GFP in proliferation conditions. In differentiation conditions NGF is repressed in both of cell. B. Elisa results showing significant differences between NGF protein levels in OBNS/PC-GFP-hNGF compared to control cells. C. hNGF signal intensity between OBNS/PC-GFP and OBNS/PC-GFP-hNGF. Biological replicates (n=3) of the aforementioned 4 cell classes were compared under proliferation and differentiation conditions.

To ensure the effectiveness of our infection protocol, the expression levels of hNGF were evaluated. Based on the results of our microarray analysis, hNGF mRNA levels are higher in OBNS/PC-GFP-hNGF respect to OBNS/PC-GFP (Figure 1 A and C). Accordingly by ELISA, we could detect higher hNGF protein in OBNS/PC-GFP-hNGF cell medium than in medium of OBNS/PC-GFP, under proliferating conditions, at day 6 (Figure 1B). Following differentiation, NGF release was repressed in both hNGF+ and WT cells, although higher level of hNGF could still be detected in OBNS/PC-GFP-hNGF (Figure 1B). The heatmap shows the effect of differentiation on the expression level of a cluster of transcripts involved in NGF signaling (Figure 1A). These results suggested that the genetic engineering of these cells to achieve the over-expression of hNGF would be a viable route for directing these cells toward a mature neuronal phenotype in view of future cell transplantation experiments in animal models of neurodegenerative diseases.

### Gene Expression Profiling of OBNS/PC-GFP, and OBNS/PC-GFP-hNGF During Proliferation and Following Differentiation

To assess correlations between our examined four cell classes, we performed gene expression profiling for differentially expressed genes between OBNS/PC-GFP (proliferated and differentiated), and OBNS/PC-GFP-hNGF (proliferated and differentiated). Biological replicates (n=3) of the aforementioned 4 cell classes were compared under proliferation and following differentiation conditions. Relative gene expression for representative probsets (250–400) in different populations was plotted using a heatmap. The proliferating OBNS/PC-GFP were distinguished from the differentiating OBNS/PC-GFP. Similarly, the proliferating OBNS/PC-GFP-hNGF were distinguished from the differentiating OBNS/PC-GFP-hNGF.

### Proliferating OBNS/PC-GFP vs. Proliferating OBNS/PC-GFP-hNGF

The analysis of transcriptome dynamics between proliferating OBNS/PC-GFP *vs.* proliferating OBNS/PC-GFP-hNGF revealed that 13 transcripts were modulated between the two cell populations (Table S1 in File S1). The modulated genes are EIF3L, CNIH2, MAPK10, ZNF585B, TUBA1A, SNORA41, GFPT2, PTK2, OR4D6, SYNPR, LHFPL3, LSAMP, and MIR9-1. While most 69% (9) of the modulated genes (EIF3L, CNIH2, MAPK10, ZNF585B, TUBA1A, SNORA41, GFPT2, PTK2, OR4D6) were up-regulated in OBNS/PS-GFP-hNGF only, 31% (4) of modulated genes (SYNPR, LHFPL3, LSAMP, and MIR9-1) were up-regulated in OBNS/PC-GFP. KEGG pathway analysis for the 9 up-regulated genes in OBNS/PC-GFP-hNGF revealed the enrichment of ErbB signaling pathway. Insufficient ErbB signaling in humans is associated with the development of neurodegenerative diseases, such as multiple sclerosis and Alzheimer’s disease [56]. In mice loss of signaling by any member of the ErbB family results in embryonic lethality with defects in organs including the lungs, skin, heart and brain. Excessive ErbB signaling is associated with the development of a wide variety of types of solid tumor. ErbB-1 and ErbB-2 are found in many human cancers, and their excessive signaling may be critical factors in the development and malignancy of these tumors [56]. The enrichment of ErbB signaling pathway in our OBNS/PC-GFP-hNGFin comparison to OBNS/PC-GFP might promote their neurogenic and glial differentiation potential which is crucial for directed therapeutic strategies against traumatic and neurodegenerative insults. Moreover, the un-modulation ErbB-1 and ErbB-2 in OBNS/PC-GFP-hNGF and OBNS/PC-GFP might decrease their cancer transformation potential.

### Differentiated OBNS/PC-GFP Vs. Differentiated OBNS/PC-GFP-hNGF

Thirteen transcripts were modulated between the differentiated OBNS/PC-GFP and differentiated OBNS/PC-GFP-hNGF (Table S2 in File S1). The modulated genes are KCNN2, KIAA0825, GK, RPS27, MEIS2, TMEFF2, WBP11, CASK, FST, RGS8, RARB, NR4A2, LRRTM2. While 23% (3 genes) of the modulated genes (KCNN2, KIAA0825, GK) were up-regulated in differentiated OBNS/PS-GFP-hNGF, 77% (10 genes) of modulated genes (RPS27, MEIS2, TMEFF2, WBP11, CASK, FST, RGS8, RARB, NR4A2, LRRTM2) were up-regulated in differentiated OBNS/PC-GFP. KEGG pathway analysis for the 3 up-regulated genes in differentiating OBNS/PC-GFP-hNGF revealed the enrichment of PPAR signaling pathway. PPARs are nuclear hormone receptors that bind peroxisome proliferators and control the size and number of peroxisomes produced by cells. PPARs mediate a variety of biological processes, and may be involved in the development of several chronic diseases, including diabetes, obesity, atherosclerosis, and cancer [58]. Enrichment of PPAR signaling pathway in OBNS/PC-GFP-hNFG might increase their cancer transformation potential as compared to OBNS/PC-GFP.

### Changes in the Gene Expression Profile of OBNS/PC-GFP and OBNS/PC-GFP-hNGF in Response to Differentiation

For OBNS/PC-GFP, 381 genes were modulated following their “shifting” from proliferation to differentiation phase of which 186 were up-regulated and 194 were down-regulated (Table S 3 in File S1). For OBNS/PC-GFP-hNGF, 556 genes were modulated of which 146 genes were up-regulated and 410 genes were down-regulated (Table S 4 in File S1). Because the differentiation of OBNS/PC-GFP and OBNS/PC-hNGF was associated with modulation of a variable number (381 for OBNS/PCs-GFP vs. 556 for OBNS/PC-GFP-hNGF) of genes, we reason that transduction of OBNS/PC-GFP with hNGF gene may modulate the differentiation potential of them [57].

### Functional Annotation Clustering of Proliferated vs. Differentiated OBNS/PC-GFP

To further highlight the effects of hNGF on the differentiation potential of OBNS/PC-GFP, we did a functional annotation for the significant genes that were identified by microarray analysis during the shifting of OBNS/PC-GFP from proliferation to differentiation phases using the web-accessible program named Database for Annotation, Visualization and Integrated Discovery (DAVID) version 2009. Clustering for the 186 up-regulated genes (Table S 5 in File S1) of OBNS/PCs-GFP using DAVID had identified 63 annotation clusters with an enrichment score ranged from 9.47 to 0.01 (Table S 5 in File S1).

The annotation cluster 9 had an enrichment score of 1.72 and included 9 groups of biological processes (BP): regulation of nervous system development (8 genes), regulation of neurogenesis (7 genes), regulation of neuron differentiation (6 genes), regulation of cell development (7 genes), negative regulation of cell differentiation (7 genes), negative regulation of neurogenesis (3 genes), negative regulation of cell development (3 genes), regulation of neuron projection development (3 genes), and regulation of cell projection organization (3 genes). Transcripts of genes involved in regulation of aforementioned 9 groups of BP comprised EPHB2, TIMP2, FOXG1, MT3, NTRK2, OMG, PBX1 were up-regulated in differentiated OBNS/PC-GFP by 1.56, 1.84, 1.43, 1.43, 1.58, 1.96, and 1.48 respectively as compared to the proliferating ones (Table S 3 in File S1). In addition, modulation of the aforementioned genes was not revealed in differentiated OBNS/PC-GFP-hNGF. This observation might indicate different differentiation potential for the two cell classes (Table S 3 in File S1).

EPHB2 signaling controls lineage plasticity of adult neural stem cell niche cells, ependymal cells and astrocytes, in the neurogenic lateral ventricle walls in the adult mouse brain. EphB2 acts downstream of Notch and is required for the maintenance of ependymal cell characteristics, thereby inhibiting the transition from ependymal cell to astrocyte [56]. The over expression of EphB2 in our differentiated OBNS/PC-GFP as compared to differentiated OBNS/PC-GFP-hNGF might indicate their less differentiated nature, and their inclination to be differentiated into ependymal cells rather than astrocyte.

The transcription factor Foxg1 is an important regulator of telencephalic cell cycles. Its inactivation causes premature lengthening of telencephalic progenitor cell cycles and increased neurogenic divisions, leading to severe hypoplasia of the telencephalon. These proliferation defects could be a secondary consequence of the loss of Foxg1 caused by the abnormal expression of several morphogens (Fibroblast growth factor 8, bone morphogenetic proteins) in the telencephalon of Foxg1 null mutants [59]. Brain-derived neurotrophic factor (BDNF) and its TrkB receptors play a central role in neuronal maturation and plasticity. The differences in expression profile of intracellular calcium responses to BDNF and ATP in subpopulations of differentiating NPCs combined with changes in the expression of BDNF and TrkB suggest cell subtype-specific alterations during early neuronal maturation [60].

The oligodendrocyte myelin glycoprotein (OMG) inhibits axon regeneration after injury in the adult mammalian central nervous system. Neural stem cells (NSC) expressed both OMG and its receptor Nogo-R1. An over-expression of OMG affected NSC expansion by reducing cell proliferation, but did not affect their differentiation into neurons. The up-regulation of OMG in differentiated OBNS/PC-GFP compared to OBNS/PC-GFP-hNGF suggests a new role for OMG during brain development as a possible regulator of neurogenesis [61]. These findings indicate a new role for OMG during brain development as a possible regulator of neurogenesis [61].

PBX1 is expressed embryonically in the telencephalon. In addition, it is expressed at high levels postnatally in the SVZ, in the migratory pathway to the olfactory bulb, and in the layers of the olfactory bulb that are the targets of these migratory neurons. SVZ proliferating cells and their neuronal progeny express PBX1 mRNA, whereas glial cells do not express detectable levels of PBX1. The expression of PBX1 in SVZ precursor cells and postmitotic neurons suggests a role for PBX1 in the generation of olfactory bulb interneurons and in mammalian neurogenesis [62].

The annotation cluster 10 had an enrichment score of 1.7 and included 16 groups of biological processes (BP): cell projection morphogenesis (8 genes), cell part morphogenesis (8 genes), cell motion (11 genes), cell morphogenesis involved in neuron differentiation (7 genes), neuron projection morphogenesis (7 genes), axon guidance (5 genes), neuron differentiation (10 genes), cell morphogenesis involved in differentiation (7 genes), neuron projection development (7 genes), axonogenesis (6 genes), neuron development (8 genes), central nervous system neuron development (3 genes), cell morphogenesis (8 genes), cell projection organization (8 genes), central nervous system neuron differentiation (3 genes), and cellular component morphogenesis (8 genes). Transcripts of genes involved in regulation of aforementioned 16 groups of BP comprised EPHA4, EPHB2, KITLG, EDNRB, FEZ1, FOXG1, FOXJ1, ID1, NRXN1, and PPAP2B were up-regulated in differentiated OBNS/PC-GFP by 1.42, 1.56, 1.5, 1.83, 1.49, 1.43, 1.89, 1.65, 1.54, and 1.47 respectively as compared to the proliferated ones (Table S 5 in File S1), and to differentiated OBNS/PC-GFP-hNGF.

Ephrin-B3 has been shown to reduce the death of endogenous NS/PC in the subventricular zone of the mouse brain without inducing uncontrolled proliferation. EphA4 receptors were expressed by spinal cord-derived NS/PC. In vivo, ephrin-B3-Fc increased the proliferation of endogenous ependymal cells and the proportion of proliferating cells that expressed the glial fibrillary acidic protein astrocytic marker in the injured spinal cord [63]. Based on this information, the over expression of EPHA4 in OBNS/PC-GFP as compared to the OBNS/PC-GFP-hNGF ones might increase their potentiality toward the expression of the glial fibrillary acidic protein astrocytic marker.

Two signaling systems mediated by EDNRB have been identified as critical players in enteric neurogenesis. Interaction between these signaling pathways controls enteric nervous system development throughout the intestine. Activation of EDNRB specifically enhances the effect of RET signaling on the proliferation of uncommitted ENS progenitors [64].

Fasciculation and Elongation Protein Zeta-1 (FEZ1) interact with DISC1 to synergistically regulate dendritic growth of newborn neurons in the adult mouse hippocampus, and this pathway complements a parallel DISC1-NDEL1 interaction that regulates cell positioning and morphogenesis of newborn neurons [65]. The expression of FoxJ1 in the brain acts on an array of target genes to regulate the differentiation of ependymal cells and a small subset of astrocytes in the adult stem cell niche. Moreover, a subset of FoxJ1(+) cells harvested from the stem cell niche can self-renew and possess neurogenic potential [66]. The tumor suppressor p53 regulates NSC proliferation and differentiation via the bone morphogenetic proteins (BMP)-Smad1 pathway and its target gene inhibitor of DNA binding 1 (Id1). p53 deficiency leads to up-regulation of Id1 which contributes to augmented proliferation and, unexpectedly, accelerated neuronal differentiation of p53(-/-) NSCs as well [67]. Decreased expression of NRXN1 in NSC resulted in changes of expression levels for the cell adhesion pathway and neuron differentiation pathway. Furthermore, astrocyte marker GFAP was significantly reduced in a time dependent manner that correlated with NRXN1 reduction. NRXN1 deletions impact several biological processes during neurodevelopment, including synaptic adhesion and neuron differentiation [68]. Ppap2b(-/-) (Lpp3(-/-)) ES cells differentiated in vitro into spinal neurons show a considerable reduction in the amount of neural precursors and young neurons formed. In addition, differentiated Lpp3(-/-) neurons exhibit impaired neurite outgrowth [69].

From the preceding discussion, the overexpression of Foxg1, PBX1, FEZ1, NRXN1 in differentiated OBNS/PC-GFP as compared to differentiated OBNS/PC-GFP-hNGF might highlight their enhanced neurogenic potential, and their implication in the generation of interneurons in the olfactory bulb.

The annotation cluster 11 had an enrichment score of 1.66 and included 3 groups of cellular components (CC): genes related to synapse (10 genes), synapse part (7 genes), and synapse (4 genes). Transcripts of genes involved in regulation of aforementioned 3 groups of CC comprised ATP1A2, CAMK2N1, CASK, GABBR1, MT3, neurexin 1, NTRK2, SYT11, and VAMP2 were up-regulated in differentiated OBNS/PC-GFP by 2.36, 1.57, 1.69, 1.72, 1,43, 1.58, 1.39, and respectively as compared to the proliferated ones (Table S 5 in File S1), and differentiated OBNS/PC-GFP-hNGF.

CASK modulates gene expression and its abundance in cultured neurons is regulated by synaptic activity. During early development, CASK was expressed in regions where neuronal progenitor cells were actively dividing, the ventricular and subventricular zones, suggesting that in addition to regulating gene expression in mature neurons, CASK may also play a role in dividing cells [70]. TrkB expression is essential for dendrite retraction and functional maturation of these neurons [71]. The overexpression of CASK and TrkB in OB/NS-GFP might indicate their enhanced neurogenic potential as compared to OBNS/PC-GFP-hNFG ones.

Clustering for the 194 down-regulated genes (Table S 6 in File S1) of OBNS/PC-GFP using DAVID had identified 71 annotation clusters with an enrichment score ranged from 9.41 to 0.00 (Table S 6 in File S1).

The annotation cluster 2 had an enrichment score of 7.12 and included 13 groups of biological processes (BP): cell division (20 genes), cell cycle (24 genes), cell cycle (31 genes), cell division (19 genes), cell cycle process (25 genes), mitotic cell cycle (19 genes), cell cycle phase (20 genes), mitosis (11 genes), M phase (15 genes), nuclear division (12 genes), mitosis (12 genes), M phase of mitotic cell cycle (12 genes), organelle fission (12 genes). The down-regulation of different genes related to cell cycle, mitosis, and cell cycle process in differentiated OBNS/PC-GFP as compared to proliferating ones might indicate the less proliferating nature of OBNS/PC-GFP following their differentiation where their genetic programs are directed toward differentiation into glial and neural progenitors.

### Functional Annotation Clustering of Proliferated vs. Differentiated OBNS/PC-GFP-hNGF

For OBNS/PC-GFP-hNGF, 556 genes were modulated of which 146 genes were up-regulated and 410 genes were down-regulated (Table S4 in File S1). Clustering analysis for the 146 up-regulated genes (Table S2, S7 in File S1) of OBNS/PC-GFP-hNGFs using DAVID where we identified 47 annotation clusters (Table S 7 in File S1).

The annotation cluster 14 showed an enrichment score of 0.86 and included 4 groups of biological processes (BP) that include genes related to synaptic transmission (6 genes), transmission of nerve impulse (6 genes), cell-cell signaling (8 genes), and neurological system process (7 genes) (Table S 7 in File S1). Transcripts of genes involved in regulation of aforementioned 4 groups BP comprised CRYAB, GNAO1, ILDR2, IL6ST, MSI1, SORBS1, THRA, ERBB4, and were up-regulated in differentiated OBNS/PC-GFP-hNGF by 3.14, 1.44, 1.52, 1.76, 1.56, 1.71, 1.5, 1.57, and 2.81 respectively as compared to the proliferated ones (Table S 4 in File S1), and to OBNS/PC-GFP.

CRYAB is one of the genes involved in cell death and survival [72], and its up-regulation in our OBNS/PC-GFP-hNGF as compared to OBNS/PC-GFP might indicate its critical role to maintain the viability and the differentiated state of OBNS/PC-GFP-hNGF in culture.

IL6ST significantly enhanced in vitro survival and promoted differentiation of human ESC-derived NP cells. It reduced caspase-mediated apoptosis and reduced both spontaneous and H2O2-induced reactive oxygen species in culture. In vitro, NP cell proliferation and the yield of differentiated neurons were significantly higher in the presence of LIF. In NP cells, LIF enhanced cMyc phosphorylation, commonly associated with self-renewal/proliferation. Also, in differentiating NP cells LIF activated the phosphoinositide 3-kinase and signal transducer and activator of transcription 3 pathways, associated with cell survival and reduced apoptosis. When differentiated in LIF+ media, neurite outgrowth and ERK1/2 phosphorylation were potentiated together with increased expression of gp130, a component of the LIF receptor complex [73]. The up-regulation of IL6ST in our OBNS/PC-GFP-hNGF might significantly promote their cell proliferation, survival, and differentiation in vitro as compared to OBNS/PC-GFP ones.

Musashi1 (Msi1) is an RNA-binding protein that is highly expressed in neural stem/progenitor cells (NS/PC) as well as in other tissue stem cells. Msi1 binds to the 3'-UTR of its target mRNAs in NS/PC, prevents their translation, and interferes with NS/PC differentiation. These results suggest that Msi1 can influence stem cell maintenance and differentiation by controlling the subcellular localization of proteins involved in miRNA biogenesis, as well as by regulating the translation of its target mRNA [74]. Our study revealed that MSi1 is up-regulated in both differentiated OBNS/PC-GFP-hNGF, and differentiated OBNS/PC-GFP in comparison to their proliferated counterparts, and was not modulated between differentiated OBNS/PC-GFP-hNGF, and differentiated OBNS/PC-GFP or proliferated OBNS/PC-GFP-hNGF, and proliferated OBNS/PC-GFP. The up-regulation of Msi1 in our differentiated OBNS/PC-GFP-hNGF, and differentiated OBNS/PC-GFP might suggest their high proliferative nature although they are in differentiated state. So this suggests that those cells still preserve a remarkable fraction of cells which maintain the pool of stem cells

The annotation cluster 21 showed an enrichment score of 0.72 and included 16 groups of biological processes (BP) that include genes related to locomotory behavior (6 genes), neuron projection development (5 genes), cell projection organization (6 genes), axon guidance (3 genes), neuron differentiation (6 genes), neuron development (5 genes), axonogenesis (3 genes), cell morphogenesis involved in neuron differentiation (3 genes), and neuron projection morphogenesis (3 genes) (Table S 7 in File S1).

Transcripts of genes involved in regulation of aforementioned 16 groups BP comprised EPHA4, FOXJ1, CALCOCO1, GNAO1, MYO6, and NRXN1 and were up-regulated in differentiated OBNS/PC-GFP by 1.63, 1.49, 1.54, 1.44, 1.49 respectively as compared to the proliferated ones (Table S 4 in File S1).

Ephrins and their Eph receptors belong to a signaling network that regulates neurogenesis. EphA4 is expressed only by neural stem cells (NSCs), and its expression maintains NSCs in an undifferentiated state. Epha4 knockdown resulted in a decrease of NSC proliferation and premature differentiation [75]. The up-regulation of Msi1 and EPHA4 in our OBNS/PC-GFP-hNGF might indicate their high proliferation nature as compared to OBNS/PC-GFP ones.

FOXJ1 is a member of the Forkhead/winged-helix (Fox) family of transcription factors, which is required for the differentiation of the cells acting as adult neural stem cells which participate in neurogenesis and give rise to neurons, astrocytes, oligodendrocytes. FOXJ1 plays an important role on neuronal production and neurogenesis in the adult brain after cerebral ischemia [76]. The present study revealed that FOXJ1 was up-regulated in differentiated OBNS/PC-GFP and OBNS/PC-GFP-hNGF by 1.89 and 1.49 folds respectively, a finding that might indicate the marked ability of OBNS/PC-GFP-hNGF, and OBNS/PC-GFP for neuronal production and neurogenesis. MYO6 may play a pivotal role in the mechanism underlying the suppressed adult neurogenesis after traumatic stress [77].

NRXN1 deletions impact several biological processes during neurodevelopment, including synaptic adhesion and neuron differentiation [78]. NRXN1 was up-regulated in differentiated OBNS/PC-GFP-hNGF and OBNS/PC-GFP by 1.74 and 1.54 fold respectively. This finding indicates the crucial role NRXN1 in neuron differentiation of our examined two cell classes.

Following differentiation of OBNS/PC-GFP and OBNS/PC-GFP-hNGF, the major markers of pluripotent such as NANOG, OCT4 [POU5F1], REX1 [ZFP42], FGF4, FOXD3, CLDN6, GDF3, DNMT3A, and CD2, were down-regulated. Genes commonly associated with a neural stem/progenitor cell fate and cycle progression: Jagged 1 (JAG1) [79], SOX2 [80], SOX4 [81], Nestin (NES) [82], the oligodendrocyte lineage transcription factor two (OLIG2) [81], the G protein-coupled receptor 56 (GPR56) [83,84], the vascular endothelial growth factor (VEGFdisintegrin and metalloproteinase domain nine (ADAM9), HAT1-, protein kinase-, DNA-activated, catalytic polypeptide (PRKDC), or RNA binding motif protein 3 (RBM3) were down-regulated in both cell types (Table S 1, 2 in File S1).

### KEGG Pathway Analysis of the Enriched Genes of Differentiated OBNS/PC-GFP vs. OBNS/PC-hNGF

To further decipher differences in the differentiation potential of OBNS/PC-GFP and OBNS/PC-GFP-hNGF, we did KEGG pathway analysis of the up-regulated 186 genes which were identified following the differentiation of OBNS/PC-GFP where we have disclosed the enrichment of MAPK signaling pathway, ErbB signaling pathway, Axon guidance, and neuroactive ligand-receptor interaction pathway, (Table S 8 in File S1). In comparison, the KEGG pathway analysis of the enriched 146 genes following differentiation of OBNS/PC-GFP-hNGF has disclosed the enrichment of Axon guidance (Figure S 2), and calcium channel, voltage-dependent, gamma subunit 7 (Table S 9 in File S1). Enrichment of functionally distinct signaling pathways between our examined two cell classes highlight different capacity for propagation and differentiation following hNGF infection with marked inclination of OBNS/PC-GFP-hNGF and OBNS/PC-GFP toward propagation, and neurogenic differentiation, respectively.

Taken together, the genetic and pathway analysis of our examined two cell classes during their proliferation and differentiation phases had revealed the variable modulation of genes and corresponding pathways. The upregulation of Foxg1, PBX1, FEZ1, NRXN1 CASK, TrkB and MAPK, ErbB, axon guidance, and neuroactive ligand-receptor interaction signalling pathway in differentiated OBNS/PC-GFP as compared to differentiated OBNS/PC-GFP-hNGF might highlight their enhanced neurogenic potential, and their implication in the generation of interneurons in the olfactory bulb. On the other hand, the up-regulation of CRYAB, GNAO1, ILDR2, IL6ST, MSI1, SORBS1, THRA, ERBB4, and enrichment of ErbB signaling pathway in OBNS/PC-GFP-hNGF as comparison to OBNS/PC-GFP might promote their cell growth, and glial differentiation potential especially their tendency toward oligodendrocyte differentiation. The un-modulation of cancer-related genes such as ErbB-1 and ErbB-2 in OBNS/PC-GFP-hNGF and OBNS/PC-GFP might decrease their cancer transformation potential.

### Phase Contrast Microscopy, Immunocytochemistry and WB Confirmation

#### Adult Human OB–NS/PC in Culture

**Figure 2:**
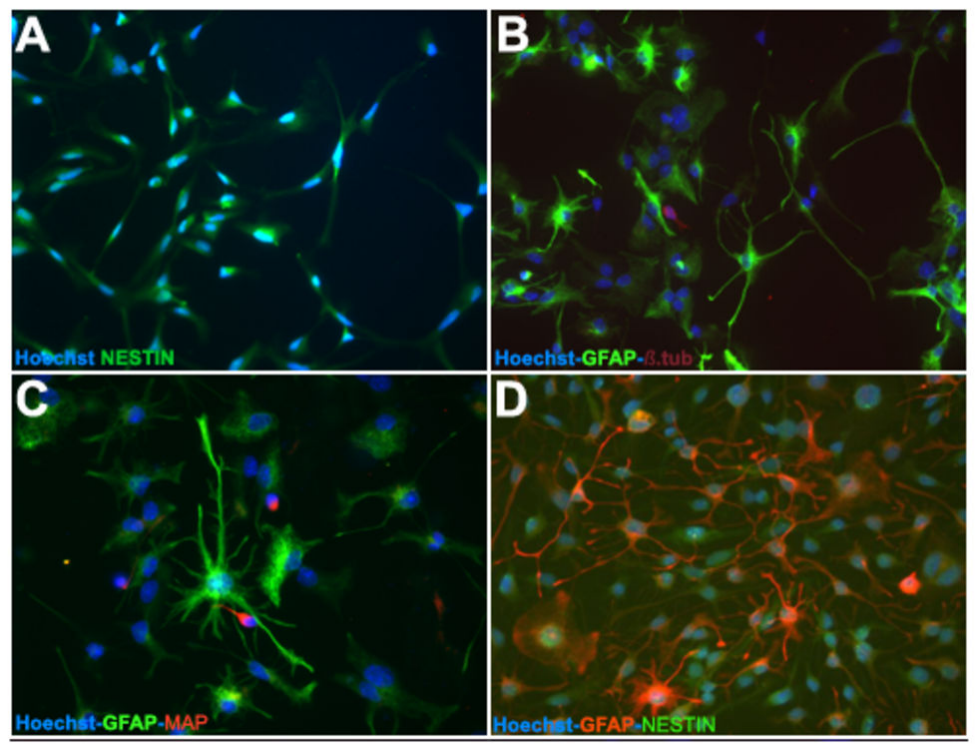
Fluorescence image (40X) of wild type OBNS/PC at day 5 post differentiation (passage 15). The OBNS/PC were stained for the NSC phenotype markers nestin (green) and GFAP (green, b, c, and red, d), MAP (red, c), β-TubulinIII (red, b). Cell nuclei were counterstained with Hoechst (blue, a-f). At the immunohistochemical level, differentiated wild type OBNS/PC exhibited positive immunoreactivity for astrocytes marker (65-75%) (B, C, D), MAP2, mature neuronal marker (8%) (C), β-TubulinIII, early neuronal marker (3%) (B).

**Figure 3:**
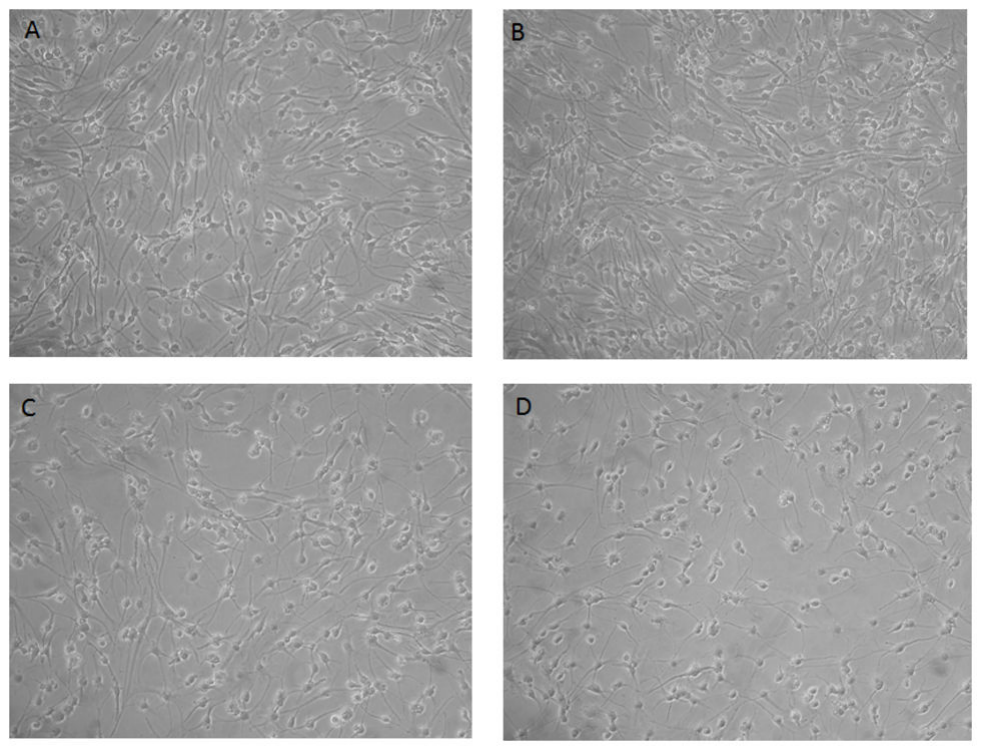
Phase contrast microscope image for OBNS/PC-GFP-hNGF (a,b), and wild type OBNS/PC (c,d) at day four of differentiation. The OBNS/PC-GFP-hNGF were more confluent and showed extensively branched processes in comparison to the wild type OBNS/PC.

To decipher the precise effects of hNGF overexpression on differentiation, we validated the microarray data regarding the effects of hNGF on the expression of the top 5 markers that were confirmed as neurogenic and glial markers by ICC and WB. Under serum-free conditions (proliferation media) and in the presence of EGF and bFGF mitogens, adult human non-transfected OBNS/PC proliferate to generate cellular clusters, primary neurospheres, with latencies ranged from 6–8 weeks, and proliferation capacity lasted for several months. Proliferating adult human wild OBNS/PC exhibited an intense immunoreactivity for nestin (Figure 2 A), none of them expressed differentiation markers: GFAP, MAP2, and O4. Differentiation assays were performed at passage 15 in the absence of EGF, bFGF and in the presence of 1% fetal calf serum supplemented with cAMP, all-trans retinoic acid. Using phase contrast microscope, the day 4-5-differentiated OBNS/PC-GFP-NGF showed extensively branched cytoplasmic processes, and were more confluent (Figure 3, A, B) in comparison to wild type OBNS/PC (Figure 3, C, D). The cell growth assay revealed that OBNS/PC-GPF-hNGF grow at a significantly faster rate to OBNS/PC-GPF (Figure S1). At the immunocytochemical level, differentiated wild type OBNS/PC exhibited positive immunoreactivity for nestin (Figure 2A), astrocytes marker (65-75%) (Figure 2 B, C, D), MAP2 mature neuronal marker (8%) (Figure 2 C), β-TubulinIII immmature neuronal marker (3%) (Figure 2 B), NG2 immature oligodendrocyte marker (8% without PDGFAA+T3, and 15% with PDGFAA+T3), and O4 mature oligodendrocyte marker was not revealed in our ICC conditions.

#### Adult Human OBNS/PC-GFP in Culture

**Figure 4:**
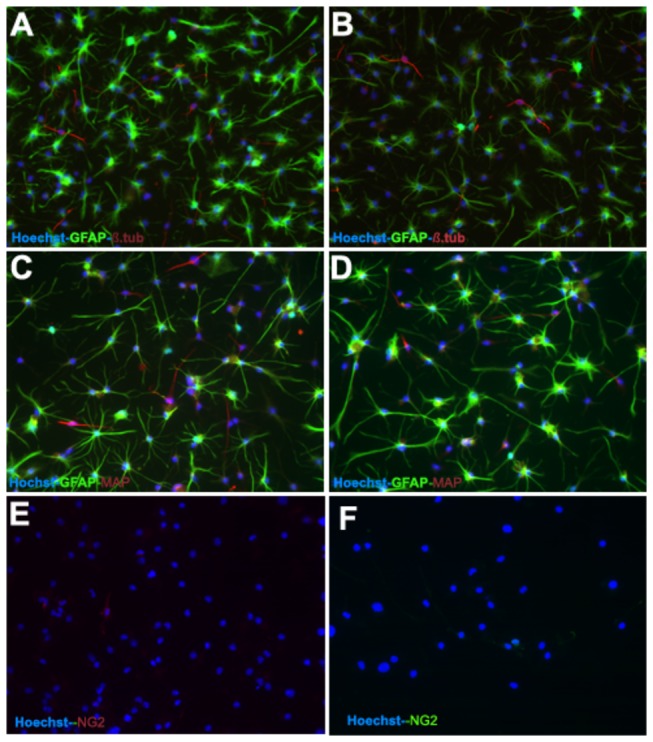
Between P7 and P10 the OBNS/PC were infected with lentivirus transducing GFP. Differentiation of OBNS/PC-GPP was applied between passage 12-15. The differentiated OBNS/PC-GFP exhibited positive immunoreactivity for GFAP astrocytes marker (60-70%) (Figure 5, A,B,C,D), MAP2 mature neuronal marker (15%) (C, D), β-TubulinIII immature neuronal marker (8%) (A,B), NG2 positive cells w/o T3 + PDGFAA (E) (7-8%), and with T3 + PDGFAA (F) (16%). The nuclei were stained blue with Hoechst.

Between P7 and P10, OBNS/PC were infected with lentivirus transducing GFP, which is one of the most common markers used to trace them following engraftment. To clarify the possible effects of such transfection protocol on proliferation and differentiation of OBNS/PC-GPP, we applied the same differentiation protocols previously described for differentiation of wild type (non-transfected) OBNS/PC. Differentiation of OBNS/PC-GFP was applied at passage 15, and differentiated exhibited positive immunoreactivity for GFAP astrocytes marker (60-70%) (Figure 4 A, B, C, D), MAP2 mature neuronal marker (15%) (Figure 4C, D), β-TubulinIII immmature neuronal marker (8%) (Figure 4 A, B), NG2 immature oligodendrocyte marker (7-8% without PDGFAA+T3 (Figure 4 E), and 16% with PDGFAA+T3 (Figure 4 F), and O4 immmature oligodendrocyte marker is not expressed in our conditions (Table 1).

**Table 1:** Differences in differentiation potential between wild OBNS/PC, OBNS/PC-GFP, and –OBNS/PC-GFP-hNGF using GFAP, MAP2, β-TubulinIII, NG2, and O4 immunocytochemistry markers.

**Cell Type**	**Control OBNS/PC**	**GFP- OBNS/PC**	**GFP-NGF-OBNS/PC**
GFAP +ve (Astrocyte marker)	65-75%	60-70%	45-55%
MAP2 +ve (Mature neurons)	8%	15%	25-30%
β-TubulinIII +ve (Immmature neurons)	3%	8%	6%
NG2+ve (Immature oligodendrocytes)	8%	7-8%	14%
NG2 +ve +PDGFAA+T3	15%	16%	25%
O4+ve (Immature oligodendrocytes)	Negative	negative	negative

#### Adult Human OBNS/PC-GFP-hNGF in Culture

**Figure 5:**
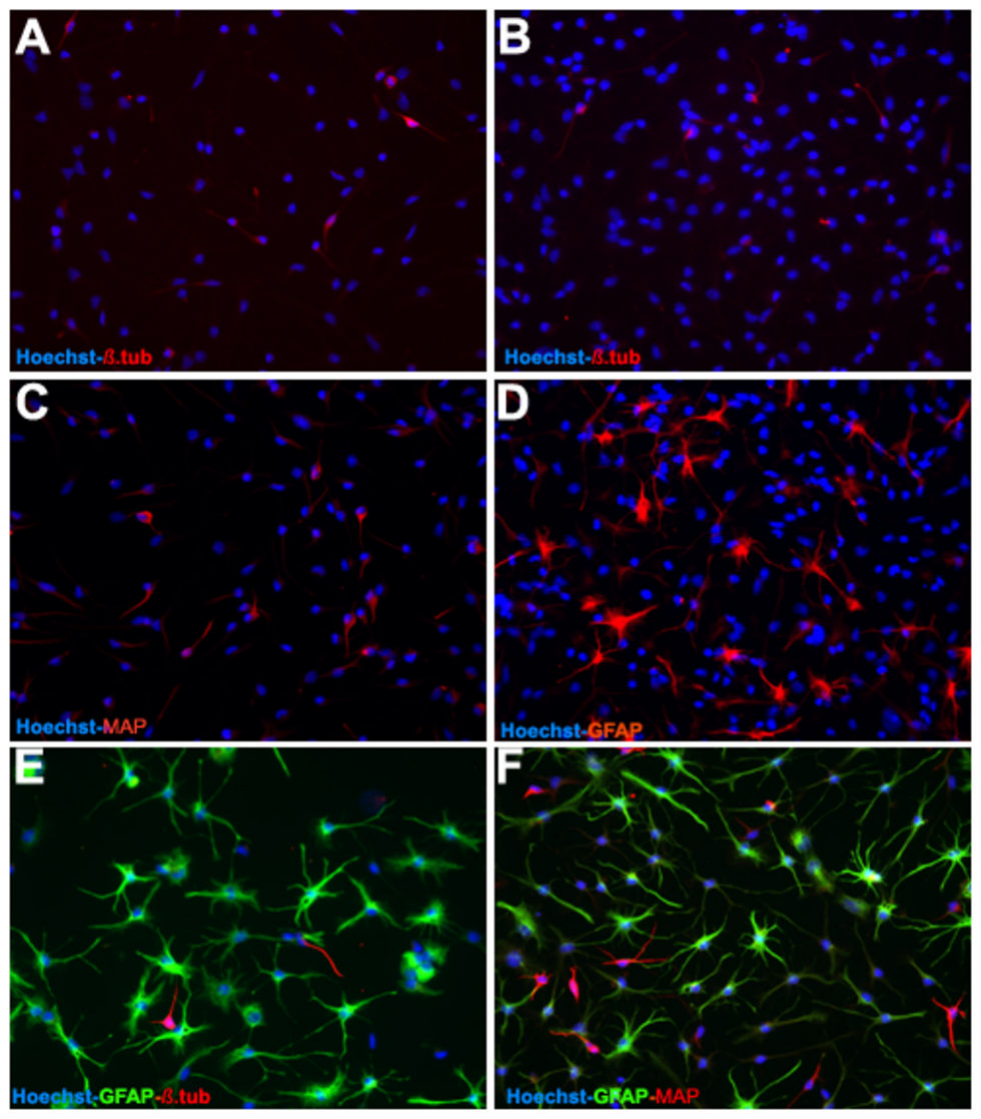
The differentiation potential of OBNS/PC-GFP-hNGF was assessed by examining their reactivity against different neuronal and glial cells molecular markers between passage 12-15. In comparison to wild type (control) OBNS/PC and OBNS/PC-GFP, differentiated OBNS/PC-GFP-hNGF exhibited positive immunoreactivity for GFAP astrocytes marker (45-55%) (D,E,F), MAP2 mature neuronal marker (25-30%) (C,D,F). β-TubulinIII, immmature neuronal marker (6%) (A,B,E). The nuclei were stained blue with Hoechst.

**Figure 6:**
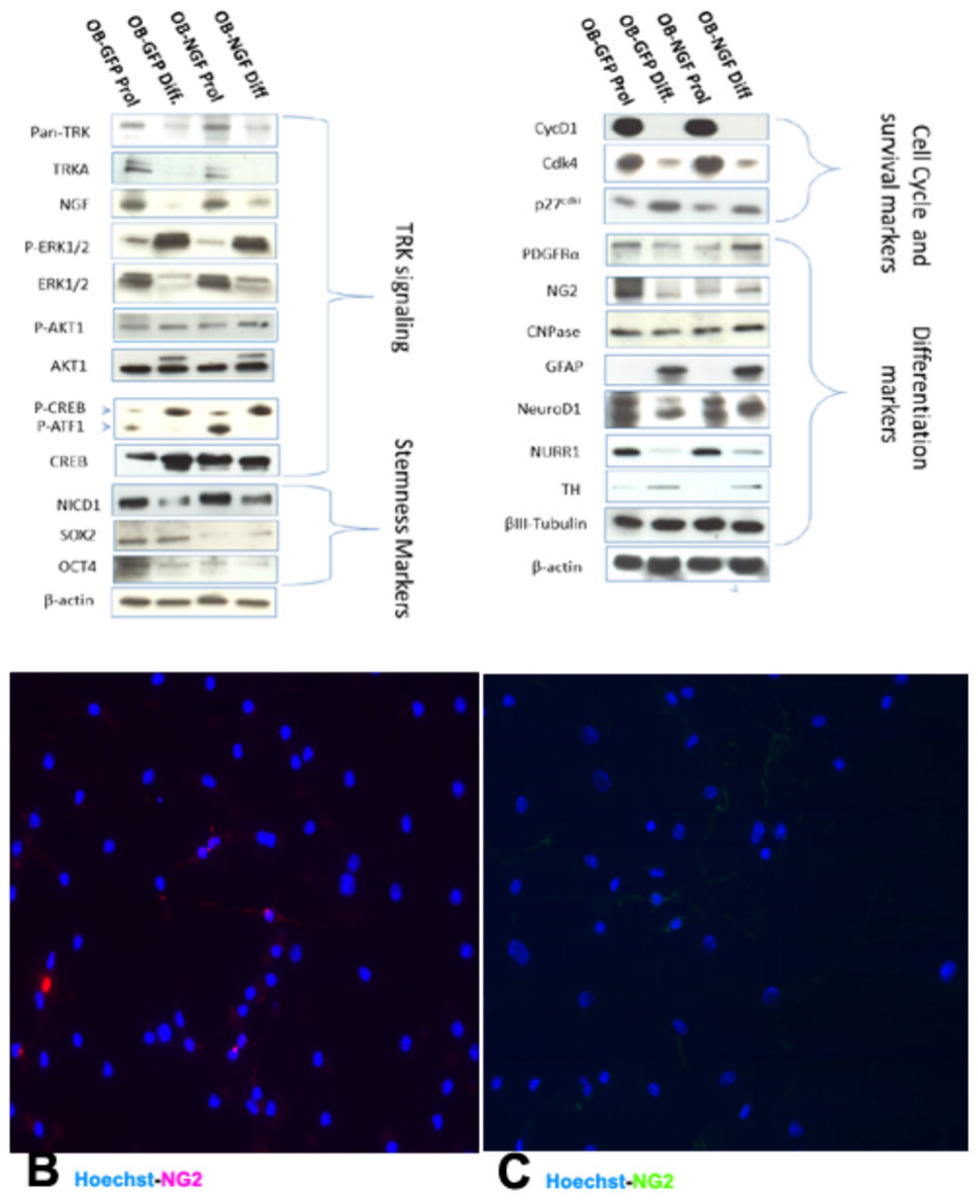
Western blot data revealed that the NGF protein levels look similar although the NGF levels were higher in differentiated OBNS/PC-GFP-NGF respect to OBNS/PC-GFP. Up- regulation of early oligodendroglia markers (PDGFRα, NG2 and CNPase) was observed in OBNS/PC-GFP-hNGF respect to OBNS/PC-GFP. Sox2 and Oct4 were up-regulated in OBNS/PC-GFP vs. OBNS/PC-GFP-hNGF. Nurr1, TH, NeuroD1 and β-TubulinIII expression were similarly expressed in the 4 cell populations. Survival genes (ERK, AKT1, CREB) were differently modulated between the 4 classes of cell populations; high-affinity Trk receptor was down modulated in differentiated cell populations vs proliferated ones. **B**. Without T3+PDGFAA, 14% of the differentiated OBNS/PC-GFP-hNGF exhibited positive immunoreactivity for NG2 oligodendrocyte marker. **C**. In the presence of T3+PDGFAA, almost 25% of differentiated OBNS/PC-GFP-hNGF were NG2 positive. The nuclei were stained blue with Hoechst.

Adult human OBNS/PC-GFP-hNGF gave rise to proliferating neurospheres, first appeared within 1 week of primary culture. The proliferating cellular clusters increased in diameters and attain 80% plate confluence within 2 weeks from the onset of culture. The neurospheres were splitted into single cells using accutase. Approximately 95% of the cells stained positive for the undifferentiated NSC marker nestin, (data not shown), and showed negative immunereactivity for GFAP, MAP2, indicating their highly proliferative and undifferentiated nature. After four days post differentiation, the OBNS/PC-GFP-NGF showed extensively branched cytoplasmic processes (Figure 3, A,B) in comparison to the wild type OBNS/PC (Figure 3, C,D). The differentiation potential of OBNS/PC-GFP-hNGF was assessed by examining their reactivity against different neuronal and glial cells molecular markers at passage 15. In comparison to wild type (control) OBNS/PC and OBNS/PC-GFP, differentiated OBNS/PC-GFP-hNGF exhibited positive immunoreactivity for GFAP astrocytes marker (45-55%) (Figure 5 D, E,F), MAP2 mature neuronal marker (25-30%) (Figure 5 C, F), β-Tubulin III imature neuronal marker (6%) (Figure 5 A, B, E), NG2 immature oligodendrocyte marker (14%, without T3+PDGFAA (Figure 6 B), and 25% with T3+PDGFAA) (Figure 6 C), and O4 mature oligodendrocyte marker was not revealed in our ICC condition (data not shown). The OBNS/PC-GFP-hNGF could be passed at least for 25 generations by mechanical dissociation and their stemness and multipotency could be maintained in serum-free medium supplemented with growth factors for at least one year. NG2 is a type of glia found in the central nervous system, which is distinct from astrocytes and oligodendrocytes [85,86]. They get their name from the expression of NG2 proteoglycan on their surface. NG2-expressing glia were believed to be the precursors of oligodendrocytes, but recent evidences suggest that the different distribution of NG2 cells is correlated to differences in physiology among cerebellar areas and reflects changes during aging [87–89].

### Western Blot (WB)

By Western blot (WB), NGF high-affinity receptor (TrkA) and pan-Trk were down modulated in differentiation conditions. hNGF protein levels reflected the result observed in ELISA (Figure 6 A). This means that OBNS/PC-GFP-hNGF were more active in releasing hNGF, that is also confirmed by extensively branched cell processes and connections with one another (Figure 3, A-D).

To highlight how overexpression of NGF influence OBNS/PC potential to differentiate into neuronal and glial subtypes, we have studied the effects of NGF over expression on modulation of major cellular key markers such as high-affinity Trk receptor and Trk signaling, stemness, survival, cell cycle and differentiation markers. Earlier reports [90] shown that the engagement of Trk receptors results in increase of extracellular signal-related kinase 1/2 activities (Erk1/2), phosphatidylinositol 3-kinase (PI3K) and phospholipase C-gamma 1 (PLC-γ1) phosphorylation, as well as cyclic AMP response element-binding protein (CREB). Several groups concurrently identified Akt1 as a downstream target of PI3K activation [91]. Phosphorylated Akt1 positively regulates CREB and NF-kB, whose mediate the expression of several pro-survival genes [92]. Initially described as a transcription factor activated by stimuli that raise intracellular levels of cAMP and lead to PKA activation [92], CREB was found subsequently to be phosphorylated also at Ser-133 on Ca^2+^ influx or growth factor stimulation or during multiple forms of synaptic hippocampal plasticity [93,94,95]. Therefore, CREB seems to act as an element of convergence and cross-talk between distinct signaling pathways, rather than as a target of one single pathway. In the present study, we assessed the regulation of activated CREB phosphorylated at Ser-133 in OBNS/PC-GFP cells versus OBNS/PC-GFP-hNGF either in proliferation or differentiation conditions. The densitometric analysis of proteins bands normalized respect to β-actin levels reported a significant increase of the p-CREB/CREB, p-ERK/ERK1/2 and p-AKT1/AKT1 ratios in differentiated cells respect to proliferated ones: 2.98, 17,82, 1,44 fold of expression respectively in OBNS/PC-GFP-NGF and 1.83, 16,23 and 1,01 fold of expression in OBNS/PC-GFP respectively. In conclusion, protein analysis of survival genes correlated with microarray data and the morphological changes and suggest that NGF over-expression exerted a beneficial neurotrophic effect during cell differentiation. In addition, we took in account the protein regulation of two pluripotent neural markers such as Sox2 and Oct4 involved in NSC self-renewal and generation of neuronal precursors [96]. Early reports have shown that constitutive expression of Sox2 inhibits neuronal differentiation and results in the maintenance of progenitor characteristics [97]. We have reported up regulation of Sox2 and Oct4 in OBNS/PC-GFP vs. OBNS/PC-GFP-hNGF in proliferation conditions. By shifting cells to differentiate, Sox2 and Oct4 proteins expression is down-modulated in OBNS/PC-GFP and undetectable levels were shown in OBNS/PC-GFP-hNGF (Figure 6 A)

Another better characterized molecular pathways shared by neural progenitor cells is the Notch signaling pathway [97]. This pathway appears to play an essential role in the maintenance of a stem/progenitor cell pool. During embryogenesis and in adulthood, expression of Notch1 or its downstream regulators, such as Hes-1, inhibits neuronal differentiation and results in the maintenance of a progenitor state. Our data, in fact, revealed a down-modulation of the Notch1 signaling highlighted by a remarkable decrease of the intracytoplasmic domain of Notch1 (NICD1) in both differentiated cells, which suggest an induction toward neuronal and glial differentiation (Figure 6 A).

Other WB data have shown a down modulation of immature oligodendrocyte markers in 5-days differentiated OBNS/PC-GFP vs. proliferatives ones, in fact, the densitometric analysis of expression levels of NG2, PDGFRα and CNPase against the protein levels of β-actin displayed a decrease to 0.17, 0.7 and 0.78 fold of expression respectively. On the contrary, we observed an increase up to 1.44, 3.21 and 1.28 fold of expression respectively for the same markers in differentiated OBNS/PC-GFP-hNGF vs proliferated ones. Previous reports observed that in the developing and neonatal rat CNS PDGFRα and NG2 can be localized in the very same punctae on the cell surface implying a close association of the two molecules [98–100]. Other experiments by Nishiyama and colleagues have shown that the co-expression of NG2 and PDGFRα is important for the proliferative response of glial progenitor cell to PDGF-AA [101]. The reduced expression of NG2 resulted in a down regulation of PDGFRα expression and the absence of NG2 leads to a defect in signal transduction, most likely due to the inability of PDGFRα and NG2 to form a complex on the surface of these cells. In conclusion, our WB data reveal the down modulation of immature oligodendrocytes markers, such as NG2 and PDGFRα, and CNPase in OBNS/PC-GFP. On the contrary, OBNS/PC-GFP-hNGF because of their higher proliferative capacity, promote up-regulation of PDGFRα and NG2 expression during the differentiation process, delaying likely the acquisition of a mature differentiation phenotype. Although during later stages of neurogenesis the PDGFRα expression appears to be mainly restricted to oligodendrocyte progenitor cells, few studies showed that some neuronal populations of the developing and adult CNS are able to express the PDGFRα [102–104]. This suggests that PDGF signaling in our context may have additional functions to its mitogenic actions for glial progenitors in the adult CNS. Moreover, the over-expression of Id1/2 observed in both differentiated cell populations by microarray along with lack of oligodendroglial fate determinants, Olig1/2, might play a crucial role in the astrocyte/oligodendrocyte fate decision.

NeuroD1 and Nurr1, the transcriptional factors expressed during neuronal dopaminergic commitment, and tyrosine hydroxylase (TH), the enzyme required for dopamine synthesis, were expressed in both cell populations and none significant modulation was observed by the NGF over-expression. This finding might indicate that the intrinsic ability to differentiate toward dopaminergic lineage is retained in both cell populations, although OBNS/PC-GFP-hNGF displayed a better neuronal morphology as confirmed by immunocytochemistry analysis (Figure 5).

Our DNA microarray data revealed that CycD1, CDK2 (cyclin-dependent kinase 2), key regulators in mammalian cell cycle, were down-regulated in differentiated OPBNS/PC-GFP as compared to proliferated ones with a fold change of 0.51 and 0.65 respectively (Table S 3 in File S1, and Table S 4 in File S1). In the present study we also examined the modulation of cell cycle proteins by WB analysis. CycD1 and Cdk4 resulted down modulated following cell differentiation of both differentiated cell populations, concurrently with the up modulation of p27 expression (Cyclin–dependent kinase inhibitor 1b) as consequence of cell cycle exit, a prerequisite for cell differentiation.

## Conclusions

In this study, we genetically modified adult human OBNS/PC to overexpress human NGF (hNGF) and green fluorescent protein (GFP) genes to provide insight about the effects of hNGF and GFP gene overexpression in adult human OBNS/PC on their in vitro multipotentiality using DNA microarray, immunophenotyping, and WB protocols. Our analysis revealed that OBNS/PC-GFP and OBNS/PC-GFP-NGF differentiation is a multifaceted process involving changes in major biological processes as reflected in alteration of the gene expression levels of crucial markers such as cell cycle and survival markers, stemness markers, and differentiation markers. The differentiation of both cell classes was also associated with modulations of key signaling pathways such MAPK signaling pathway, ErbB signaling pathway, and neuroactive ligand-receptor interaction pathway for OBNS/PC-GFP, and Axon guidance, and calcium channel, voltage-dependent, gamma subunit 7 for OBNS/PC-GFP-hNGF as revealed by GO and KEGG. OBNS/PS-GFP-NGF express gene markers that predict higher cell survival and proliferation potential vs. OBNS/PC-GFP. The OBNS/PC-GFP-NGF produced more NGF during the proliferation phase only, with suppression of NGF secretion following differentiation. PDGFRα and NG2 protein expression was up regulated by WB in differentiated OBNS/PC-GFP-hNGF respect to differentiated OBNS/PC-GFP along with significant morphological changes observed by ICC. Taken together these findings reveal the trophic influence exerted by NGF on NG2-oligodendroglial precursors and its ability to promote neuronal growth and branching.

## Supporting Information

File S1 Supporting tables.
Table S1 in File S1: Modulated genes between OBNS/PC-GFP-hNGF proliferation (class 1) and WT OBNS/PC-GFP proliferation (class 2).
Table S2 in file S1: Modulated genes between OBNS/PC-GFP-hNGF differentiation (class 1) and WT OBNS/PC-GFP differentiation (class 2).
Table S3 in file S1: Genes which are differentially expressed among WT OBNS/PC-GFP differentiation (class 1) vs. WT OBNS/PC-GFP proliferation (class 2).
Table S4 in file S1: Genes which are differentially expressed among OBNS/PC-GFP-hNGF differentiation (class 1) vs. OBNS/PC-GFP-hNGF proliferation (class 2).
Table S5 in file S1: Functional Annotation Clustering using DAVID for the 186 up-regulated genes of OBNS/PC-GFP had identified 63 annotation clusters with an enrichment score ranged from 9.47 to 0.01.
Table S6 in file S1: Clustering for the 194 down-regulated genes of OBNS/PC-GFP using DAVID had identified 71 annotation clusters with an enrichment score ranged from 9.41 to 0.00.
Table S7 in file S1: for OBNS/PC-GFP-hNFG, 556 genes were modulated of which 146 genes were up-regulated and 410 genes were down-regulated (Table S4). Clustering analysis for the 146 up-regulated genes of OBNS/PC-GFP-hNGF using DAVID where we identified 47 annotation clusters.
Table S8 in file S1: KEGG pathway analysis of the up regulated 186 genes which were identified following the differentiation of OBNS/PCs where we have disclosed the enrichment of MAPK signaling pathway, ErbB signaling pathway, Axon guidance, and neuroactive ligand-receptor interaction pathway.
Table S9 in file S1: in comparison, the KEGG pathway analysis of the enriched 146 genes following differentiation of OBNS/PC-GFP-hNGF has disclosed the enrichment of Axon guidance, and calcium channel, voltage-dependent, gamma subunit 7.
(RAR)

Figure S1 **Cell Growth assay.** OBNS/PC-GFP-hNGF shows a significant higher rate of cell growth in two subclones in comparison to OBNS/PC-GFP.
(PPT)

Figure S2 **Axon Guidance Pathway.** The relative expression of differentially expressed genes for the Axon guidance pathway between the four cell classes was plotted using a heatmap.
(PPTX)
