# Correction: Over-Expression of hNGF in Adult Human Olfactory Bulb Neural Stem Cells Promotes Cell Growth and Oligodendrocytic Differentiation

**DOI:** 10.1371/journal.pone.0125885

**Published:** 2015-04-16

**Authors:** The PLOS ONE Staff

Fig 5E in this article reuses an image from another *PLOS ONE* article by the same author without the attribution needed to comply with the Creative Commons Attribution License. The correct attribution for the image is: Fig 2B from the article Marei HES, Ahmed A-E, Michetti F, Pescatori M, Pallini R, Casalbore P, et al. (2012) Gene Expression Profile of Adult Human Olfactory Bulb and Embryonic Neural Stem Cell Suggests Distinct Signaling Pathways and Epigenetic Control. PLoS ONE 7(4): e33542. 10.1371/journal.pone.0033542

**Fig 5:**
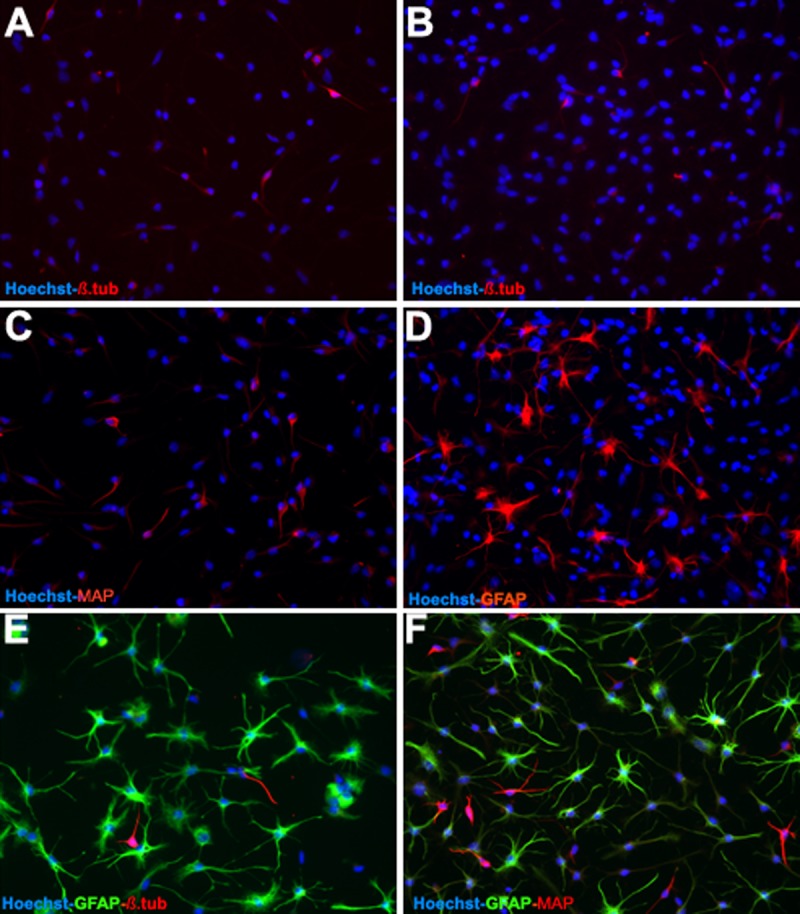
The differentiation potential of OBNS/PC-GFP-hNGF was assessed by examining their reactivity against different neuronal and glial cells molecular markers between passage 12–15. In comparison to wild type (control) OBNS/PC and OBNS/PC-GFP, differentiated OBNS/PC-GFP-hNGF exhibited positive immunoreactivity for GFAP astrocytes marker (45–55%) (D,E,F), MAP2 mature neuronal marker (25–30%) (C,D,F). β-TubulinIII, immmature neuronal marker (6%) (A,B,E). The nuclei were stained blue with Hoechst.
