# Supplementary figures and images for: Over-Expression of hNGF in Adult Human Olfactory Bulb Neural Stem Cells Promotes Cell Growth and Oligodendrocytic Differentiation
Source: PLoS One. 2013 Dec 19;8(12):e82206. doi: 10.1371/journal.pone.0082206 (PMC3868548; doi:10.1371/journal.pone.0082206)

## Slide 1
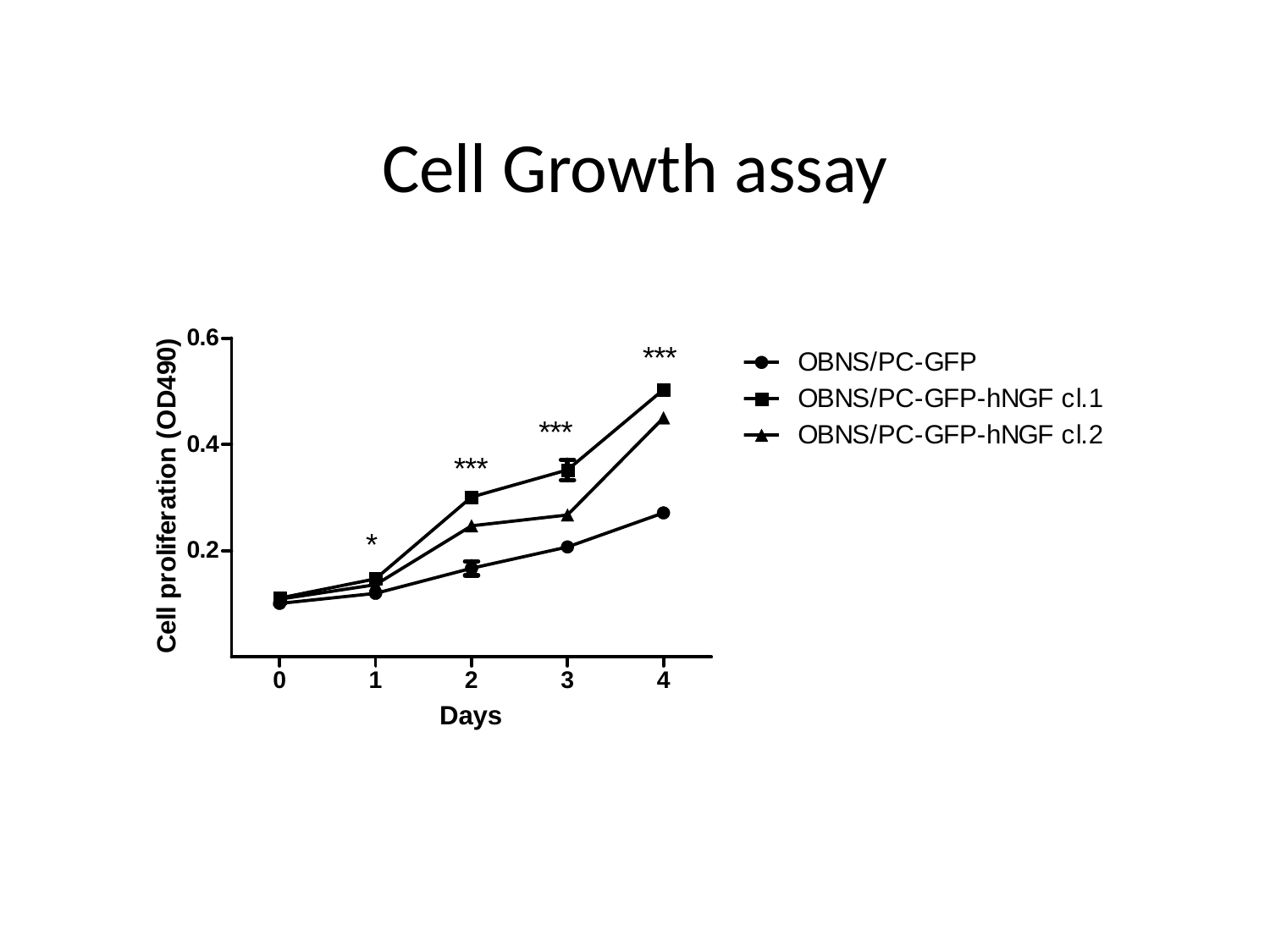

# Cell Growth assay

Supplement: Figure S1 — Cell Growth assay. OBNS/PC-GFP-hNGF shows a significant higher rate of cell growth in two subclones in comparison to OBNS/PC-GFP. (PPT) [file pone.0082206.s002.ppt]

## Slide 1
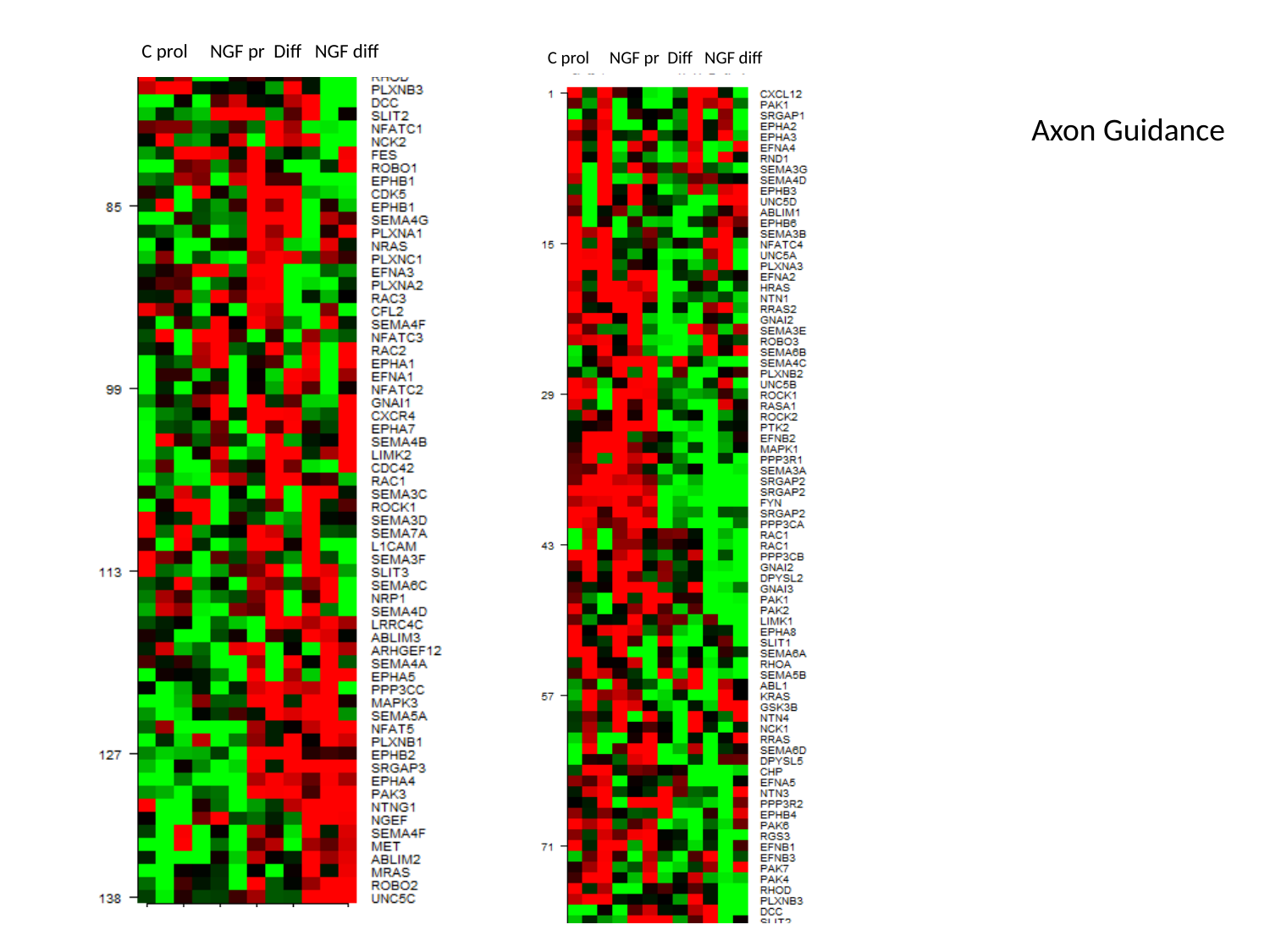

C prol NGF pr Diff NGF diff
C prol NGF pr Diff NGF diff
Axon Guidance

Supplement: Figure S2 — Axon Guidance Pathway. The relative expression of differentially expressed genes for the Axon guidance pathway between the four cell classes was plotted using a heatmap. (PPTX) [file pone.0082206.s003.pptx]
